# Strong anisotropic enhancement of photoluminescence in WS_2_ integrated with plasmonic nanowire array

**DOI:** 10.1038/s41598-021-89136-0

**Published:** 2021-05-12

**Authors:** Chunrui Han, Yu Wang, Weihu Zhou, Minpeng Liang, Jianting Ye

**Affiliations:** 1 Institute of Microelectronics, Chinese Academy of Sciences, Beijing, 100029 China; 2 Device Physics of Complex Materials, Zernike Institute for Advanced Materials, University of Groningen, Nijenborgh 4, 9747 AG Groningen, The Netherlands

**Keywords:** Nanophotonics and plasmonics, Two-dimensional materials

## Abstract

Layered transition metal dichalcogenides (TMDCs) have shown great potential for a wide range of applications in photonics and optoelectronics. Nevertheless, valley decoherence severely randomizes its polarization which is important to a light emitter. Plasmonic metasurface with a unique way to manipulate the light-matter interaction may provide an effective and practical solution. Here by integrating TMDCs with plasmonic nanowire arrays, we demonstrate strong anisotropic enhancement of the excitonic emission at different spectral positions. For the indirect bandgap transition in bilayer WS_2_, multifold enhancement can be achieved with the photoluminescence (PL) polarization either perpendicular or parallel to the long axis of nanowires, which arises from the coupling of WS_2_ with localized or guided plasmon modes, respectively. Moreover, PL of high linearity is obtained in the direct bandgap transition benefiting from, in addition to the plasmonic enhancement, the directional diffraction scattering of nanowire arrays. Our method with enhanced PL intensity contrasts to the conventional form-birefringence based on the aspect ratio of nanowire arrays where the intensity loss is remarkable. Our results provide a prototypical plasmon-exciton hybrid system for anisotropic enhancement of the PL at the nanoscale, enabling simultaneous control of the intensity, polarization and wavelength toward practical ultrathin photonic devices based on TMDCs.

## Introduction

Two-dimensional semiconducting transition metal dichalcogenides (TMDCs) have recently attracted considerable interest because of tunable bandgap, tightly bound excitons, and highly tunable excitonic properties^1^, enabling applications in various photonic and optoelectronic devices ranging from infrared to visible spectra, such as light-emitting diodes^2–4^, photodetectors^5,6^, photovoltaics^7^, modulators^8,9^, nanoscale quantum devices^10–12^, etc. To be a light emitter, an important property is a linear polarization, which is highlighted in areas like ultrasensitive sensing, super-resolution imaging, medical diagnosis, and optical communications^13–20^. PL of TMDCs is intrinsically linearly polarized under linear excitation, because of the coherent superposition of excitonic states in +/− *K* valleys at the corners of Brillouin zones. However, due to the Coulomb exchange interaction and intervalley scattering, valley coherence is severely suppressed at room temperature, resulting in random polarization in practice. To recover the valley coherence, resonant nanocavities have been proposed to either generate exciton–polariton quasiparticles^21,22^ or accelerate the electron–hole recombination rate to surpass quantum decoherence^12^. Despite these efforts, it remains difficult to achieve a strong PL signal with high linearity and conventional polarizers that are bulky and of high losses are still in use. To be compatible with on-chip integration, new types of anisotropic modulation of the excitonic emission are highly demanding.

Optical processes such as absorption and emission in quantum emitters can be greatly modified by using optical cavities because the interaction between light and matter closely depends on the local density of electromagnetic modes^23,24^. The enhancement occurs when the interaction between EM modes and emitters overcomes the damping in the system, such as MoS_2_/WS_2_ with nanodisks, dimer nanoantennas, and bull’s eye-shaped Bragg reflectors due to Purcell effect^25–28^, and Fano resonance in the hybrid of MoS_2_-bowtie nanoantenna array^29^. To further emphasize the optical anisotropy, metallic nanowires are more advantageous, which enable tailoring multiple parameters of light emission in a single device, e.g. wavelength, polarization, phase, and direction^30–37^. Regarding the polarization, the anisotropy arises from generally the form-birefringent effect of subwavelength nanowire arrays, which is characterized as a reflection of the TE polarization and the transmission of the TM polarization and determined by the aspect ratio of a nanowire^38,39^. Whereas, intriguing processes of plasmonic resonance and coherent interference that could also lead to strong anisotropy have not been properly treated. Hence, the radiative behaviors of a wire array-TMDCs hybrid system certainly deserve to be investigated comprehensively.

Here, we demonstrate strong anisotropic enhancement of the PL in a plasmon-exciton hybrid system consisting of a plasmonic nanowire array and WS_2_. For the indirect bandgap transition around 750 nm, up to 7 (4.2) folds enhancement was achieved with the PL polarization perpendicular (parallel) to the long axis of the nanowire. The enhancement arises from the Purcell effect which is attributed to the coupling of WS_2_ with localized (guided) surface plasmon modes in nanowire arrays of different periods. Although we observed enhancement for both TE and TM polarizations, the magnitudes of the enhancement are different, leading to a high degree of linearity. For the direct bandgap transition, a high degree of linearity was also observed because of the enhancement/suppression of the PL in TE/TM polarization directions, *i*.*e*. plasmon-enhanced coherent scattering of the TE polarization in the forward direction and the high reflection of the TM polarization in the backward direction. The degree of the linearity is found to depend on the wire width and period of the array while it is less influenced by the polarization of the excitation laser. Our results demonstrate the unique anisotropic light-matter interactions in a highly confined TMDC-metasurface device, which offer the possibility of manipulating the intensity, polarization, and wavelength of the scattered light in one single device and provide guidelines for establishing the ultrathin optical devices with multi-functionalities.

## Results

### Device configuration

**Figure 1:**
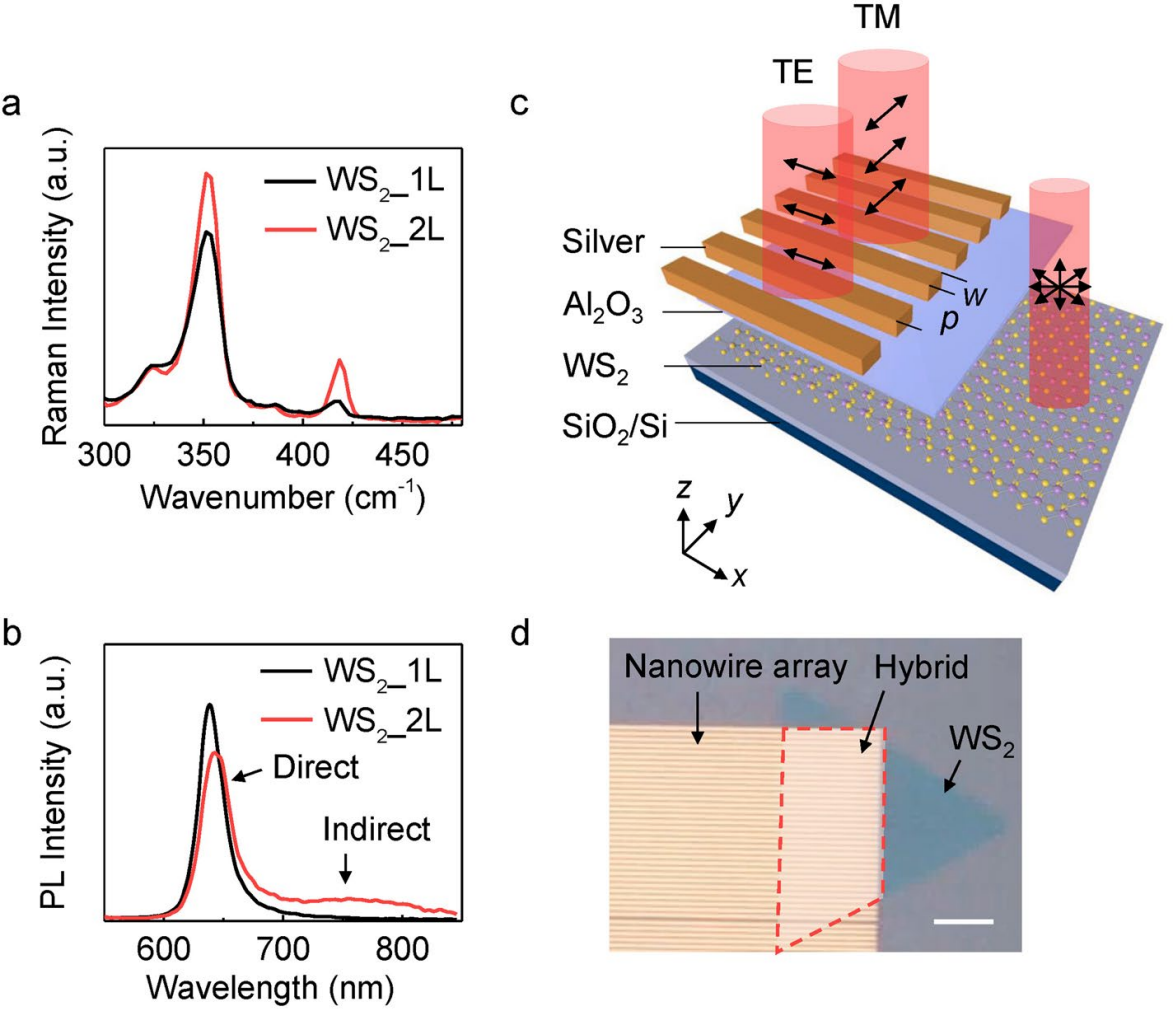
Device configuration of the WS_2_-Ag wire array hybrid nanostructure. (**a**) Raman spectra of mono- and bilayer WS_2_. (**b**) PL spectra of mono- and bilayer WS_2_. (**c**) 3D schematic view of the hybrid device consisting of silver nanowire array on top of WS_2_ separated by a thin layer of Al_2_O_3_. (**d**) Optical microscopy image of the WS_2_-Ag wire hybrid nanostructure. The scale bar is 6 μm.

WS_2_ flakes were grown by chemical vapor deposition on silicon wafers with thermally grown SiO_2_ of 285 nm (Supplementary note 1). The crystal is identified by the *E*′ ($\it {\text{E}}_{2g}^{1}$) and $\it {\text{A}}_{1}^{\prime }$(*A*_1g_) Raman bands around 350 and 418 cm^−1^ (Fig. 1a and Figure S1) for mono- (bi-) layer, respectively^40^. The layer numbers are characterized through the PL spectra, in which only the direct bandgap transition around 636 nm can be observed in the monolayer WS_2_ (black curve in Fig. 1b) while an additional broad emission appears around 750 nm (1.65 eV) corresponding to the indirect bandgap transition of a bilayer WS_2_ (red curve in Fig. 1b)^41,42^. For the bilayer WS_2_ grown by chemical vapor deposition, the intensity of the PL at 640 nm is as strong as that of the monolayer. This is probably due to the partial coverage of the second layer, leaving a mixture of monolayer and bilayer (Supplementary note 5). To establish a practical device, a 20 nm thick Al_2_O_3_ layer was deposited to protect the WS_2_ from dopants in the ambient atmosphere. Nanowire arrays with varying period *p* and wire width *w* were fabricated subsequently on top using electron-beam lithography followed by silver metallization (Fig. 1c). WS_2_ flakes are partially covered by the nanowire array to define regions with and without photonic structures for comparison, where the WS_2_-Ag hybrid region is indicated by a red dashed box (Fig. 1d).

### Anisotropic enhancement behaviors of the PL in the direct and indirect bandgap transitions

#### Experimental characterization of the anisotropic enhancement

**Figure 2:**
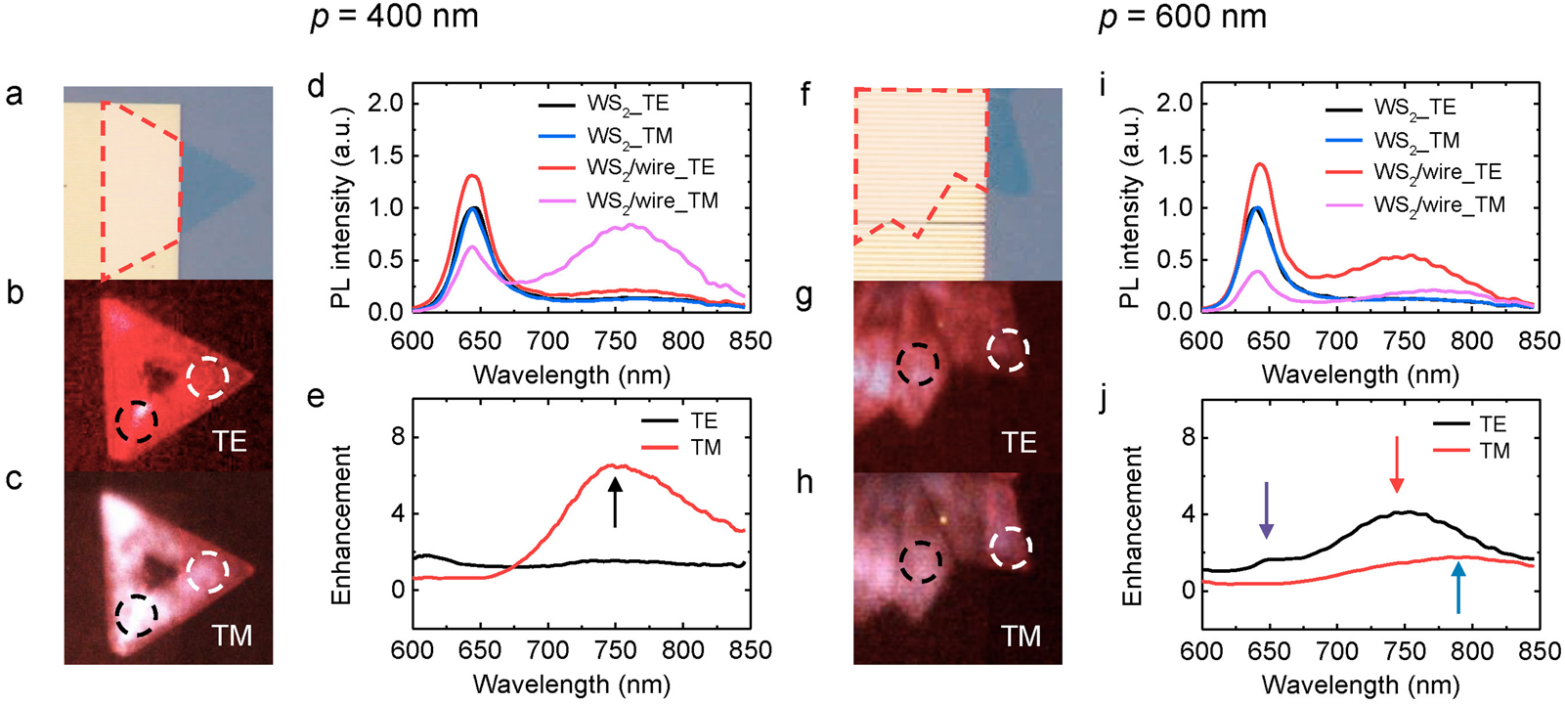
Anisotropic enhancement of the PL emission in WS_2_-Ag wire hybrid nanostructures. (**a**) Optical image of the hybrid nanostructure. WS_2_ covered by the nanowire array (*p* = 400 nm, *w* = 140 nm) is enclosed by the red dashed lines. (**b**)–(**c**) The corresponding PL images for TE and TM polarizations. The dashed circles indicate the positions of the spectral measurement. (**d**) Normalized PL spectra of the WS_2_ bilayer with (red and pink curves) and without (black and blue curves) silver nanowire array for TE and TM polarizations respectively. (**e**) PL enhancements of the hybrid nanostructure relative to the pristine WS_2_ for TE (black curve) and TM (red curve) polarizations, respectively. (**f**)–(**j**) The corresponding results for the hybrid nanostructure with *p* = 600 nm and *w* = 300 nm.

After patterning nanowire arrays, the hybrid devices were firstly characterized by polarization-resolved PL imaging (Supplementary note 2). Briefly, samples were excited by a 450 nm light-emitting diode and the polarization of the PL emission was resolved by rotating a polarizer before the CCD camera. For the *p* = 400 nm wire array, the optical image is shown in Fig. 2a where a triangular-shaped WS_2_ (blue region) is partly covered by the metasurface (yellow region). PL images of the TE and TM polarized light are shown in Fig. 2b,c, respectively. The region covered by the array is slightly brighter than that of the pristine WS_2_ for TE (Fig. 2b) but significantly brighter for TM (Fig. 2c). Note that the CCD camera showed slightly anisotropic responses between TE and TM polarizations, therefore one should NOT compare the color (i.e. intensity) between TE and TM images. Nevertheless, within each polarization image, one can roughly evaluate the enhancement of the PL intensity.

To quantify the enhancement, polarization-resolved PL spectra were taken in regions of pristine WS_2_ and hybrid WS_2_-Ag wire array respectively (Supplementary note 3). The samples were excited by a 532 nm laser with the incident polarization fixed in the TE direction. The PL emission was collected in the TE and TM directions by rotating the analyzer to be parallel and perpendicular to the long axis of the nanowire, respectively. PL spectra of the pristine bilayer WS_2_ are shown by the black and blue curves in Fig. 2d. The coincidence between TE and TM implies that the PL emission is almost randomly polarized due to the valley depolarization mechanism, such as Coulomb exchange interaction and intervalley scattering at room temperature^43^. A drastic change between TE and TM is observed when the nanowire array is fabricated on top as shown by the red and pink curves in Fig. 2d. For the direct bandgap transition around 640 nm, the PL intensity is 1.3/0.6 times enhanced for TE/TM, respectively (Fig. 2d). In contrast, for the indirect bandgap transition around 750 nm, both TE and TM are amplified. Specifically, the enhancement of the PL relative to the pristine WS_2_ is 7 for TM and 1.5 for TE as shown by the red and black curves in Fig. 2e. The enhancements are different at different spectral positions, implying that the underlying mechanisms involving the plasmon-mediated excitonic emission are different at different optical bandgap transitions.

For *p* = 600 nm wire arrays, the optical image of the WS_2_-Ag wire hybrid nanostructure is shown in Fig. 2f. The region covered by the nanowire array is brighter than that of the pristine WS_2_ in the TE polarized image (Fig. 2g) whereas it becomes significantly darker for the TM polarized image (Fig. 2h). The color contrast between the regions with and without the metasurface can be further verified by the PL spectral measurement. For the TE polarized emission, the intensity relative to that of the pristine WS_2_ is 1.4 and 4.2 times enhanced for the direct and indirect bandgap transitions (the purple and red arrows in Fig. 2j), consistent with the brighter color in Fig. 2g. In contrast, for the TM polarized emission, the intensity of the PL in the hybrid region is only 39% of the pristine WS_2_ for the direct bandgap transition (pink curve in Fig. 2i), resulting in the darker PL image as shown in Fig. 2h. The larger suppression of the TM polarization leads to the larger anisotropy between TE and TM for the 600 nm period wire array than that of the 400 nm (red and pink curves in Fig. 2d,i). For the indirect bandgap transition, the PL is greatly enhanced in the TE polarization direction, which is perpendicular to that observed in the 400 nm period wire array, implying that the period of the nanowire array is essential to control the direction of the polarization.

We’d like to emphasize that with significant improvement in polarization, the total intensity of the emission does not decay but increases due to the energy transfer. This is in contrast to the conventional passive birefringent crystal, through which the light of TE polarization is much more blocked than TM polarization thus the total intensity keeps descending due to the scattering and absorption of the crystal. In this regard, our nanowire array is NOT just a simple polarizer.

#### The mechanism for anisotropic enhancement of the indirect bandgap transition

**Figure 3:**
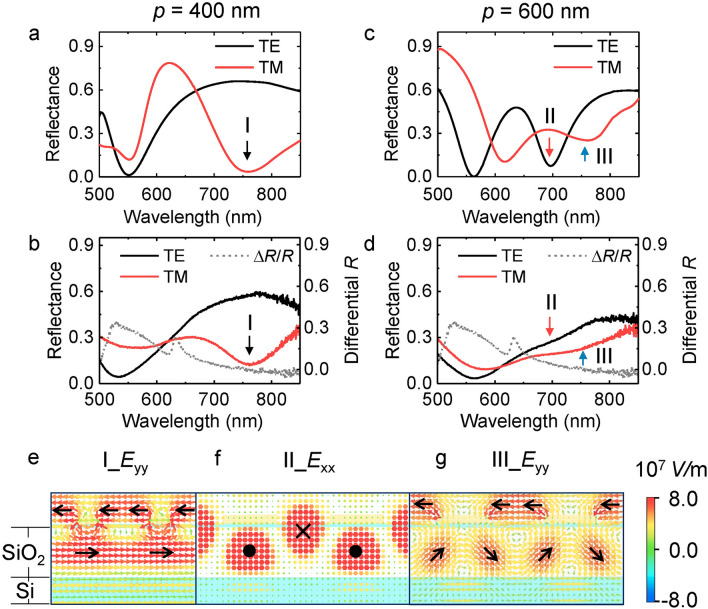
Polarization resolved white light spectra. (**a**)–(**b**) Reflectance spectra of the WS_2_-Ag wire hybrid nanostructure with *p* = 400 nm in simulation (**a**) and experiment (**b**) for TE and TM polarizations, respectively. (**c**)–(**d**) The corresponding results for the hybrid nanostructure with the period of the nanowire array 600 nm. Differential reflectance peaks ~ 630 nm [dashed grey curves in (**b**) and (**d**)] indicate the absorption of the WS_2_ monolayer. (**e**)–(**g**) Distributions of the electric fields at the *x* = 0 nm cutting plane for resonance modes I-III respectively. Black arrows indicate the directions of electric dipoles.

To understand the anisotropic enhancement behaviors observed above, optical responses of the nanowire arrays are simulated by a commercial finite-difference time-domain algorithm (FDTD Solution, Lumerical) for *x* and *y* polarization incidences. For the array of *p* = 400 nm, *w* = 140 nm, we find a pronounced reflectance dip around 750 nm (mode I) for the TM polarization as indicated by the black arrow in Fig. 3a, which is consistent with the experimental results shown in Fig. 3b. This dip overlaps well with the indirect bandgap transition, hence could be the main reason for significant PL enhancement (7x) in the TM polarization direction (black arrow in Fig. 2e). The dip is intrinsically a localized surface plasmon mode whose electric fields are densely localized around the edge of nanowires (Fig. 3e) exhibiting strongly enhanced fields and a high local density of states. The coupling of excitons with the confined plasmonic fields accelerates the decay rate of the spontaneous emission, causing a brighter PL image in the 400 nm period nanowire array (Fig. 2c). Moreover, the driving field is oscillating in the TM direction, thereby polarizing the PL emission in the same direction.

For *p* = 600 nm wire arrays, we find a reflectance dip around 700 nm (mode II) for the TE polarized light in both numerical simulation and experiment (red arrows in Fig. 3c,d). Its electric field distribution (Fig. 3f) indicates the energy is mainly concentrated in dielectric layers. The fields are polarized along the wire with opposite phases in a unit cell, resulting in a pronounced magnetic resonance around indirect excitons that accounts for the 4.2 folds enhancement of the PL emission in the TE polarization direction (red arrow in Fig. 2j). We identify mode II as a plasmonic waveguide mode rather than a localized surface plasmon of individual wire^44^, because the electromagnetic fields are confined in dielectric layers between the top metal and the bottom silicon instead of metal surfaces whose distributions follow closely the period of the wire array. Compared to mode I, the loss of this mode is smaller because of vanishing dissipation from the displacement currents of the metal due to the opposite phases of electric dipolar resonances in the dielectric layer. However, the mode volume is larger, therefore the enhancement is slightly weaker (4.2x) compared with mode I (7x). For the TM polarization, a reflectance dip appears around 750 nm (blue arrows in Fig. 3c,d) which is indicative of a resonance mode III, providing the driving force for the enhancement of the TM polarized emission (the blue arrow in Fig. 2j). The electric dipolar resonance on the top surface of wire arrays is quite similar to that of mode I, but the magnitude is only 1/4 (Fig. 3g), hence the enhancement of the PL emission is relatively small (1.8x).

Combining the experimental and simulation results, we can now understand the strong anisotropic enhancement of the indirect bandgap transition achieved in the bilayer WS_2_-silver nanowire hybrid nanostructure. The enhancement is attributed to the coupling of excitons with the plasmonic modes of the nanowire array through which the spontaneous emission rate, as well as the quantum yield, is greatly boosted in the bilayer WS_2_. The degree of the enhancement depends on the intensity of local fields, the mode volume, and the system loss. Among the three modes, mode I is a localized plasmon mode with maximum field intensity and minimum mode volume, hence it enables the largest PL enhancement (7x). Mode II is a plasmonic waveguide mode whose mode volume is much larger than that of mode I but the loss is smaller due to the magnetic resonance, hence the PL enhancement remains large (4.2x). Mode III belongs to a plasmonic mode whose field distribution is similar to that of mode I but the intensity of the local field is much weaker. In addition, the mode volume is larger because of the larger period, therefore leading to the smallest PL enhancement (1.8x). The polarization direction of the enhancement is consistent with the oscillation direction of the electric dipolar resonance in the nanowire array which can be either parallel (mode II) or perpendicular (mode I and III) to the nanowires.

#### The mechanism for anisotropic enhancement of the direct bandgap transition

As indicated by the experimental results in Fig. 2d,i, the anisotropy for the direct bandgap transition is due to the enhancement of the PL in the TE polarization direction and the suppression of the PL in the TM polarization direction (Fig. 2d,i). However, as demonstrated in this section, the TE enhancement cannot be accounted for *only* by the plasmonic effect. Rather, the interference of the periodic nanowire array plays an important role.

**Figure 4:**
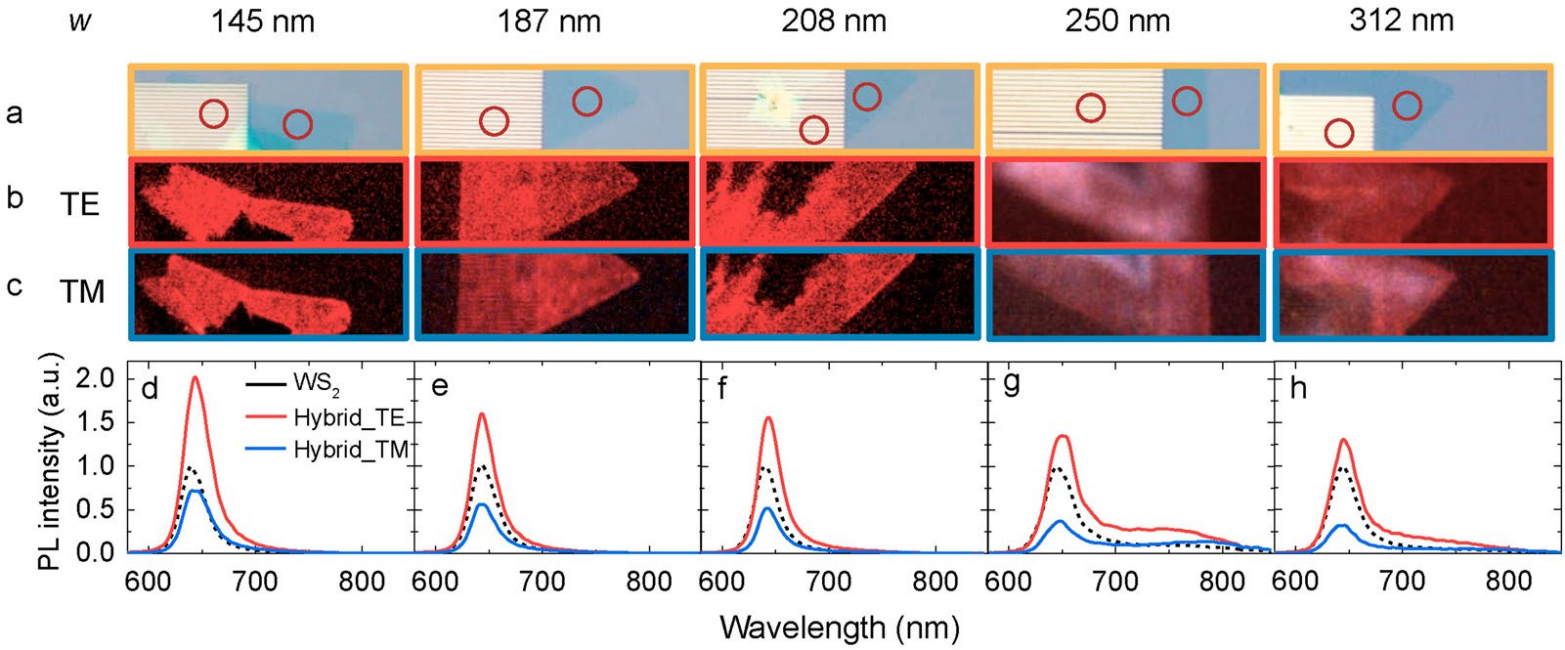
Anisotropic enhancement of the direct bandgap emission as a function of the wire width. (**a**) Optical images of the WS_2_-Ag wire hybrid nanostructures. From left to right, the width of the nanowire is 145, 187, 208, 250 and 312 nm, respectively. (**b**)–(**c**) Corresponding PL images for TE and TM polarizations. (**d**)–(**h**) Corresponding PL spectra for the pristine WS_2_ (black dashed curves), WS_2_-Ag wire hybrid nanostructures in TE (red solid curves) and TM (blue solid curves) polarization directions.

Before discussing the mechanism, we’d like to confirm the robustness of the experimental observation by varying the wire width while fixing the array period at 600 nm (optical images in Fig. 4a). PL images indicate that the WS_2_-wire hybrid region is brighter in the TE polarization direction (Fig. 4b) and darker in the TM polarization direction (Fig. 4c). Moreover, with increasing the wire width from 145, 187, 208, 250 to 312 nm, the degree of the enhancement relative to the pristine WS_2_ (dashed black curve in Fig. 4d–h) decreases from 2.0/0.72, 1.6/0.56, 1.56/0.52, 1.35/0.37 to 1.30/0.32 for the TE/TM polarized light (red/blue curves in Fig. 4d–h), implying that the wire width is an effective parameter in tailoring the intensity and anisotropy of the PL emission: while narrower wires are more efficient to enhance the PL along the wire, wider wires are more suitable to suppress the PL in perpendicular to the long axis of the wire.

**Figure 5:**
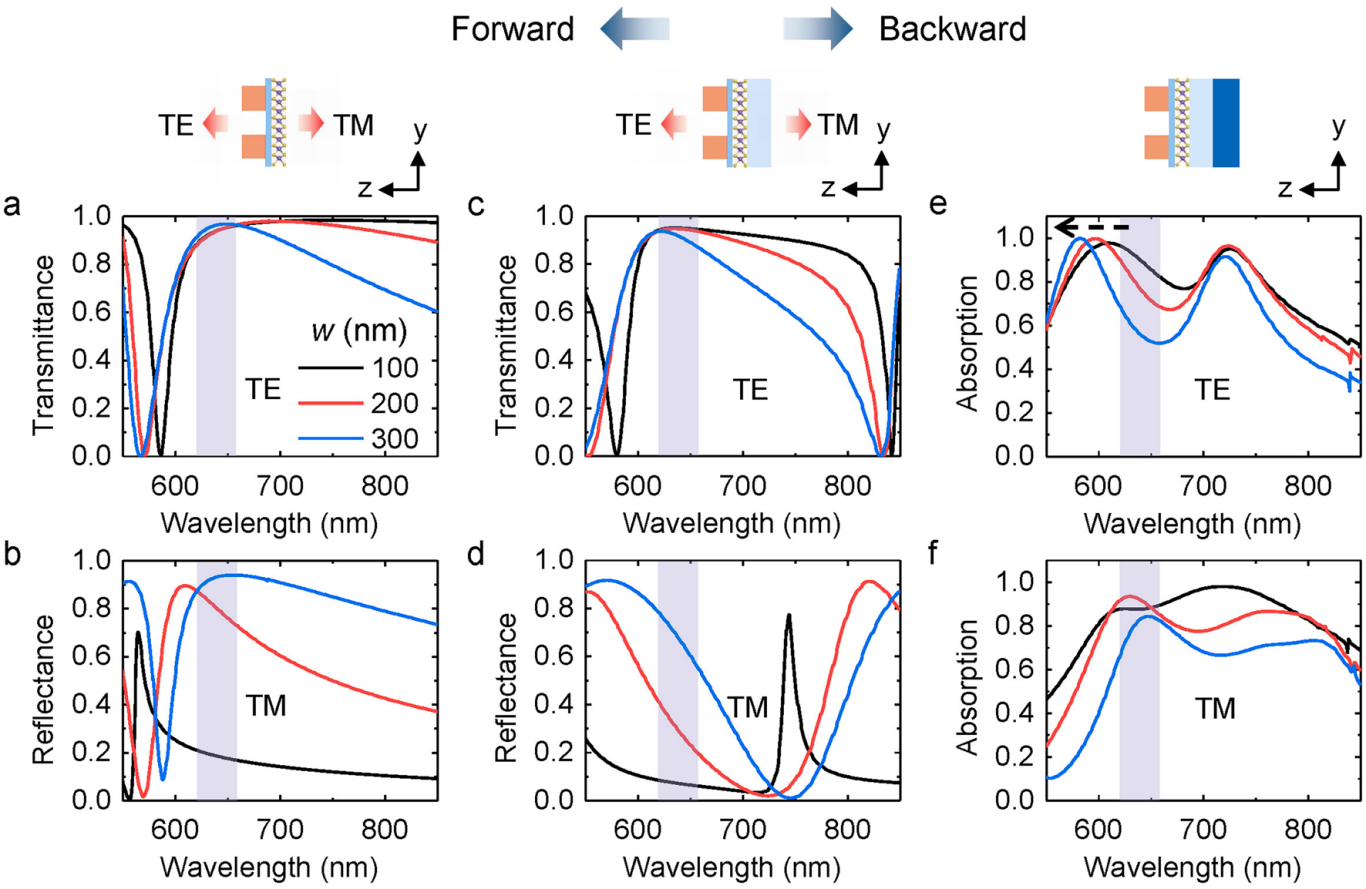
Polarization resolved spectra as a function of the wire width by simulations. (**a**)–(**b**) Transmittance of the TE polarized light and reflectance of the TM polarized light under the forward incidence for the nanowire array on Al_2_O_3_ substrate with *p* = 600 nm, *w* = 100, 200, 300 nm respectively. (**c**)–(**d**) The corresponding spectra of the nanowire array on Al_2_O_3_/SiO_2_ substrate. (**e**)–(**f**) Absorption spectra of the TE and TM polarized light under the backward incidence for the nanowire array on Al_2_O_3_/SiO_2_/Si substrates.

Now we turn to numerical simulations where the dielectric substrates (Al_2_O_3_, SiO_2_ and silicon layers) are added in a step-by-step fashion. To substantiate the experiment in Fig. 4, we choose nanowire arrays with *p* = 600 nm, *w* = 100, 200 and 300 nm in Fig. 5. For the forward incidence, that is, the light propagates at the positive z-direction, the transmittance of the TE polarized light around the wavelength of the direct bandgap transition (marked by the purple strips in Fig. 5) is high as shown in Fig. 5a,c for the nanowire array on Al_2_O_3_ and Al_2_O_3_/SiO_2_ respectively. Moreover, the reflectance of the TM polarized light increases step by step with the wire width, denoted by the black, red, blue curves as shown in Fig. 5b and d. It suggests that the nanowire array allows high transmission of the TE polarized PL in the forward direction and a tunable reflection of the TM polarized PL in the backward direction, consistent with the near field distributions of the nanowire array in *E*_xx_ and *E*_yy_ directions (Figure S2).

To be detailed, in Fig. 5e, absorption spectra of the nanowire array on Al_2_O_3_/SiO_2_/Si substrates indicate that plasmonic resonances appear around 600 nm. These peaks locate on the blue side of the spectral wavelength of the direct bandgap transition, offer feasible excitation and energy transfer between nanowires and WS_2_ excitons, leading to the enhancement of the PL emission in the TE polarization direction^45,46^. With increasing the wire width, the peak shifts to the shorter wavelength as indicated by the dashed black arrow in Fig. 5e, hence the spectral overlap between plasmons and excitons decreases, resulting in the descending trend of the PL enhancement in the TE polarization direction as indicated by the red curves in Fig. 4d–h. For the TM polarization, broadband absorption of plasmonic resonance appears around the direct bandgap transition (Fig. 5f) which is possible to accelerate the spontaneous emission rate. However, the emission in the TM polarization is mainly reflected by the nanowire array and the reflection increases with the wire width, resulting in the suppression of the PL in the forward direction and the descending trend of the PL intensity with the wire width (blue curves in Fig. 4d–h).

However, note that in Fig. 5e the direct band emission does not coincide with the plasmonic resonance in TE polarization. Especially for *w* = 300 nm, the emission approaches the valley of the absorption spectra, calling for an alternative mechanism to explain the TE enhancement. Since the nanowire array resembles a diffraction grating, the inevitable result is the constructive interference at the angle *θ* with *p*sin *θ* = m*λ*, where *θ* is the angle between the grating surface and the emission direction of light, *p* is the period of the grating and *λ* is the emission wavelength^47^. Light collected in the normal direction corresponds to *θ* = 90° which is an interference maximum, thus the emission in the TE polarization is enhanced as shown in Fig. 4b.

As a result, the strong anisotropy of the direct bandgap transition is attributed to the zeroth-order constructive interference of the nanowire array in addition to the active plasmonic resonance on the blue side of the emission wavelength in the TE polarization direction as well as the tunable reflectance of the nanowire array in the TM polarization direction.

#### Polarization modulation through the anisotropic enhancement of the PL

**Figure 6:**
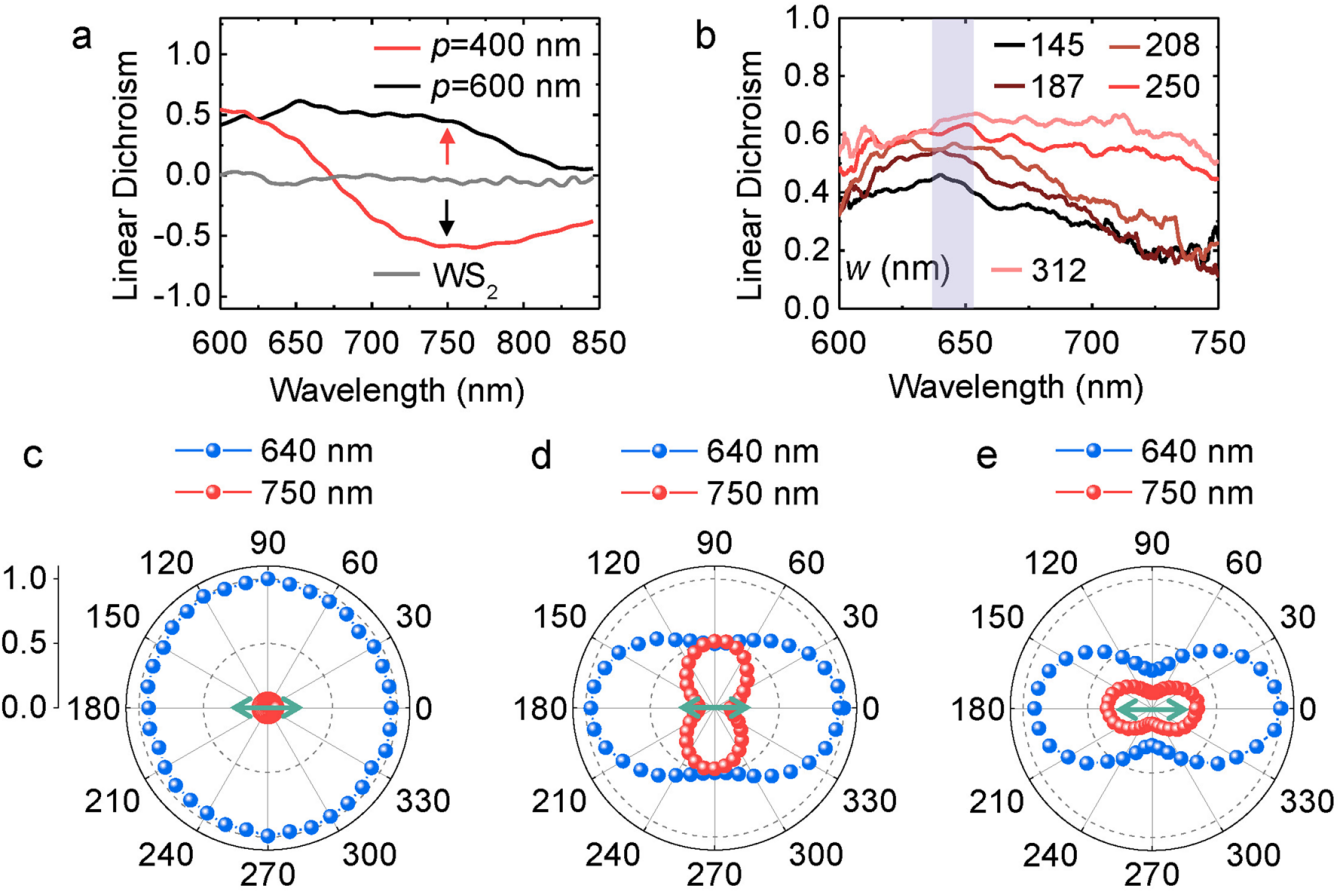
Linearity of the direct and indirect bandgap transitions modulated by the plasmonic nanowire array. (**a**) Linear dichroism of the PL emission for pristine bilayer WS_2_ (grey curve) and WS_2_-Ag hybrid nanostructures with *p* = 400 (red curve) and 600 nm (black curve) respectively. (**b**) Linear dichroism of WS_2_-Ag hybrid nanostructure with *p* = 600 nm as a function of the wire width. (**c**)–(**e**) Normalized PL peak intensity as a function of the detection angle for pristine bilayer WS_2_ (**c**) and hybrid nanostructures with *p* = 400 (**d**) and 600 nm (**e**), respectively. Blue/red dots indicate the emission of direct/indirect bandgap transitions, respectively. Here, zero corresponds to the center and 1.0 to the outermost dashed circle. Green arrows indicate the long axis of the nanowire.

To quantify the PL anisotropy as a function of the period and wire width, linear dichroism (LD) calculated as *LD* = (*I*_TE_ − *I*_TM_)/(*I*_TE_ + *I*_TM_) is shown in Fig. 6. For the indirect bandgap transition, in contrast to the diminishing LD in pristine WS_2_, a positive LD of 0.5 is achieved in the nanowire array with *p* = 600 nm, *w* = 300 nm for the indirect bandgap transition (red arrow in Fig. 6a). Additionally, a large negative LD ~  − 0.6 is observed for the nanowire array with *p* = 400 nm, *w* = 140 nm around 750 nm (black arrow in Fig. 6a). It suggests that the period of the nanowire array is able to tune the sign of the linear dichroism. The good linearity can be attributed to the distinct coupling of excitons with plasmons along the two orthogonal polarizations, leading to different degrees of enhancement of the PL in TE and TM directions.

For the direct bandgap transition, the maximal linearity is always observed in the nanowire array with *p* = 600 nm because this period is comparable with the emission wavelength *λ* ~ 636 nm thus satisfies the condition of the constructive interference *p* ~ *λ*, thereby leading to the most remarkable enhancement in the TE polarization direction among different periods of the nanowire array (red curves in Figure S3a,d). This is consistent with the highest transmittance of the TE polarized light in the forward direction among three types of periods of the nanowire array (red curves in Figure S4a,b,e,f). At the same time, the emission in the TM polarization direction is low (red curves in Figure S3b,e) because of the high reflectance in the backward direction (red curves in Figure S4c,d,g,h). Moreover, the wire width is a key parameter to tune the linearity of the PL emission of the direct bandgap transition since the dominating plasmon mode and the reflectance of the nanowire array have a close dependence on the wire width (Fig. 5). As a result, LD increases from 0.43 to 0.64 by increasing the wire width from 145 to 312 nm as shown in Fig. 6b.

To show intuitively the extraordinary polarization modulation through the anisotropic enhancement of the nanowire array, PL peaks as a function of the detection angle for indirect (red dots) and direct (blue dots) bandgap transitions are characterized for the pristine bilayer WS_2_ and WS_2_-Ag hybrid nanostructures with the periods of 400 and 600 nm as shown in Fig. 6c–e. For the indirect bandgap transition, PL is randomly polarized in the pristine WS_2_ (Fig. 6c), then becomes linearly polarized in perpendicular (Fig. 6d) or parallel (Fig. 6e) to the long axis of the wire for *p* = 400 and 600 nm wire array respectively. For the direct bandgap transition, PL transforms from random to linear with the polarization fixed along the wire despite that the period of the wire array is different. In summary, large linearity is obtained for both indirect and direct bandgap transitions in bilayer WS_2_. The former arises from the strong anisotropic enhancement of the PL in both TE and TM polarization directions dominated by either the plasmonic or waveguide mode of the metal wire array. The latter is attributed to the enhancement/suppression of the PL in the TE/TM polarization directions based on the enhanced transmission/reflection of the nanowire array in orthogonal polarizations.

#### Anisotropic emission as a function of the polarization angle of the excitation

**Figure 7:**
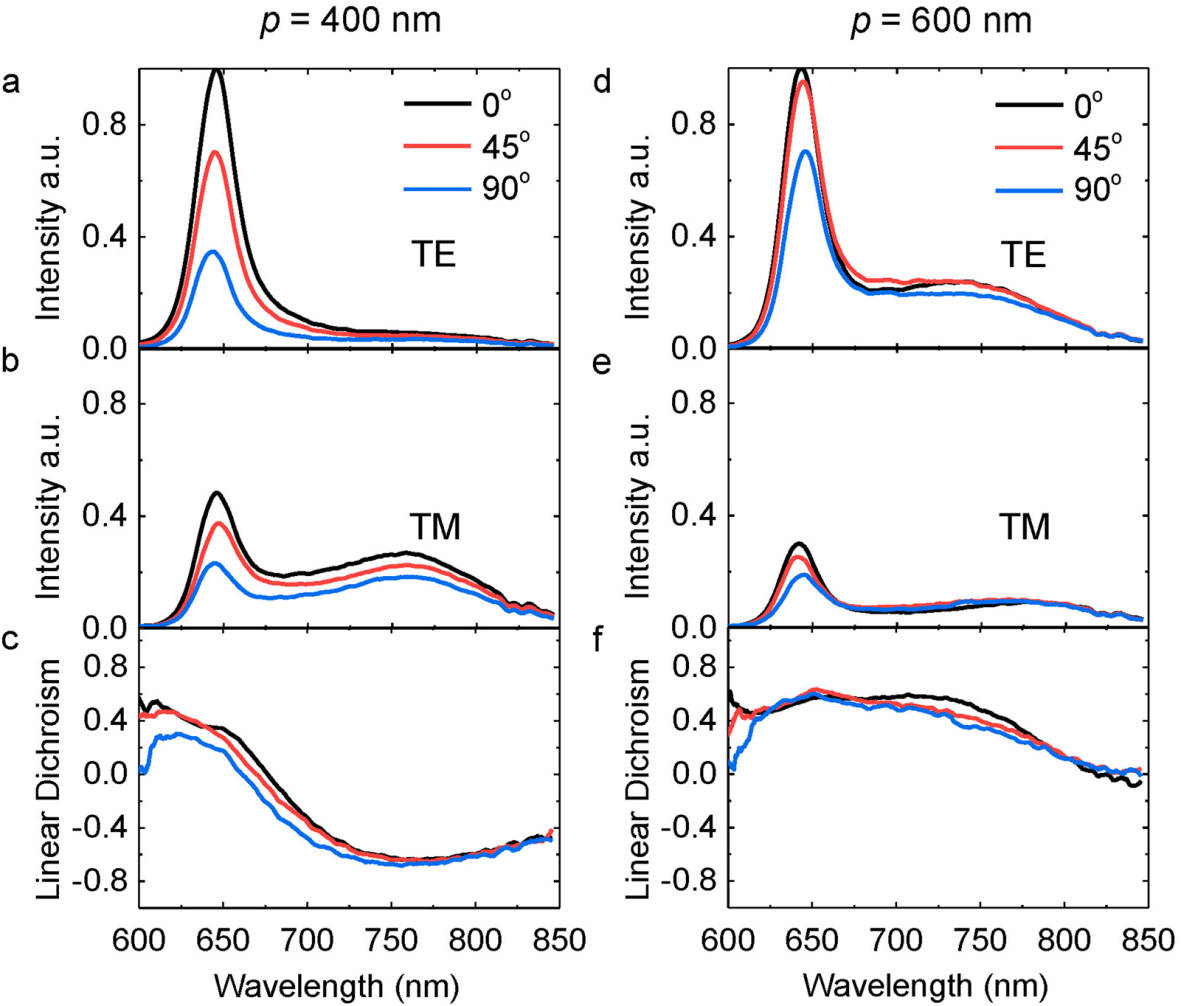
Anisotropic PL emission as a function of polarization angles of the laser excitation. Normalized intensity of the PL in the TE (**a**, **d**) and TM (**b**, **e**) polarization directions for the incident polarization angle at 0° (black curve), 45° (red curve), and 90° (blue curve), respectively. (**c**), (**f**) The corresponding linear dichroism. The periods of nanowire arrays in the hybrid WS_2_-Ag nanostructures are 400 and 600 nm for the left and right columns, respectively.

In the above, the polarization of the incident laser is fixed along the wire. What will happen if we change the incident polarization direction? For the WS_2_-Ag wire hybrid nanostructure with *p* = 400 nm, the normalized intensity of the PL in the TE/TM polarization direction is 1/0.48, 0.7/0.37 and 0.35/0.23 for the direct bandgap transition and 0.059/0.27, 0.050/0.23, 0.034/0.18 for the indirect bandgap transition when the incident polarization angle increases from 0°, 45° to 90° (Fig. 7a,b). It suggests that the intensity of the PL decreases when the incident polarization relative to the long axis of the wire is rotated from parallel (0°) to perpendicular (90°). This is probably due to the different excitation efficiency at different incident angles. The excitation is the strongest at 0° incidence due to the maximum absorption as manifested by the deeper reflection dip at ~ 532 nm as shown by the black curve in Fig. 3b. The absorption decreases with increasing the angle via 45° to 90° because the reflectance increases as manifested by the red curve in Fig. 3b resulting in the descending trend of the PL emission. Since both TE and TM polarizations decrease, the linear dichroism keeps almost the same at different excitation angles as shown in Fig. 7c.

For the hybrid nanostructure with *p* = 600 nm, a descending trend as a function of the incident polarization angle can also be observed in both TE and TM polarizations (Fig. 7d,e). But the difference of the PL intensity between 0° and 90° is smaller than that of the 400 nm period wire array (Fig. 7a,b) because the difference of the absorption at the excitation wavelength (532 nm) between TE and TM polarizations is smaller (Fig. 3d). The linear dichroism keeps almost the same for different incident polarization angles (Fig. 7f), suggesting that the degree of the linearity of the PL emission is determined by the geometry of the wire array rather than the polarization angle of the laser excitation.

## Discussion

Conventional optical modulators suffer from large volume, single function and passive modulation, thus are hard to be directly applied to photonic circuits. Here the highly confined metasurface-TMDCs hybrid systems provide a new route toward the emerging flat optics, where the intensity, polarization and wavelength of light can be tuned by tailoring the direction and magnitude of the plasmon-exciton coupling. Such a hybrid system is not limited to WS_2_ samples. Take mode II as an example, it would be red shifted from 550 to 850 nm when the period of the nanowire array increases from 400 to 800 nm (Figure S5). The spectra cover almost the whole range of bandgaps of normal TMDC mono- and few layers, for instance, 620, 650, 750 and 800 nm for WS_2_, MoS_2_, WSe_2_, MoSe_2_ monolayers respectively. Hence, it is highly promising that various TMDCs with different optical bandgap transitions can be controlled by the plasmonic nanowire array.

It is noteworthy that mechanisms of the intensity enhancement and the anisotropy of the PL are different at different wavelengths. The exciton-plasmon coupling dominates the PL enhancement around 750 nm. Significant enhancements of the PL (7 folds in TM for the 400 nm period wire array and 4.2 folds in TE for the 600 nm period wire array) are attributed to the plasmonic modes I and II (Fig. 3), which provide localized and guided plasmonic fields, respectively, to increase the spontaneous emission rate of the PL in specific polarization directions. Hence, the anisotropy of the PL can be ascribed to the different degrees of enhancement between TE and TM polarizations. However, for the emission around 640 nm, we attribute the enhancement to both the coherent scattering of the nanowire array and the exciton-plasmon coupling. The enhancement in the TE polarization direction is moderate (less than 2 folds), as shown by the red curves in Fig. 4d–h. Because the emission wavelength is comparable to the period of the nanowire array thus the constructive interference takes place. Moreover, the plasmonic mode of the nanowire array (represented as the reflectance dip around 580 nm for the TE polarization in Fig. 3c,d) located on the blue side of the PL emission wavelength may contribute to the enhancement. We have found that the blue shift of this mode, as indicated by the dashed black arrow in Fig. 5e, coincides well with the descending trend of the PL intensity in the TE polarization direction upon increasing the wire width (Fig. 4d–h).

**Figure 8:**
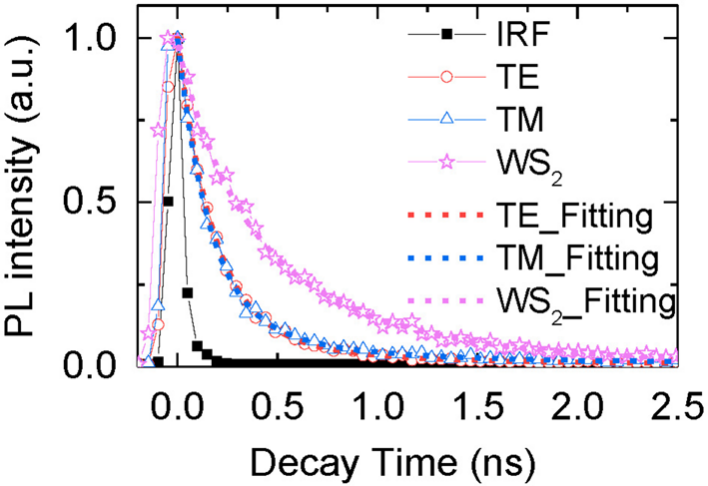
Time and polarization-resolved PL spectra of the bare WS_2_ (pink star curve) and the hybrid nanostructure. PL lifetimes of the TE and TM polarizations of the hybrid nanostructure are indicated by the red dot (TE) and blue triangle (TM) curves. Data are fitted by two-component exponentials (dashed curves with the corresponding color).

To provide more evidence of the exciton-plasmon coupling for the PL enhancement ~ 640 nm, time-resolved photoluminescence is measured to determine the PL decay time of WS_2_ integrated with/without the nanowire array (Supplementary note 4). Silver nanowire array, with 600 nm in period and 170 nm in width, is patterned on monolayer WS_2_ (Figure S6a). The polarization-resolved lifetimes of the excitonic emission for the WS_2_-Ag wire hybrid nanostructure and the bare WS_2_ are shown in Fig. 8. The hybrid structure exhibits a shorter lifetime than the bare WS_2_. Specifically, the decay process is ~ 2 times faster when the WS_2_ is integrated with the nanowire array (Table 1). Also, the decay time is quite similar between TE and TM for the hybrid nanostructure, which is probably due to the almost equal absorption for both polarizations (red and blue arrows in Figure S6b). The reduced lifetime can be explained as the plasmonic speed-up effect^48^. The plasmonic fields increase the recombination rate between electrons and holes in the WS_2_, causing the increase of the spontaneous emission rate and hence the PL intensity. Similar decay time between TE and TM implies that the PL enhancement is effective to both polarizations. As a result, the anisotropy of the PL emission at 640 nm should be originated from the preferred scattering direction of the nanowire array, *i*.*e*. TE polarization in the forward direction (Fig. 5a,c) and the TM polarization in the backward direction (Fig. 5b,d).

**Table 1:** PL lifetime of WS_2_ monolayer with/without the integration of the nanowire array (*p* = 600 nm, *w* = 170 nm). The lifetime and fractional amplitude^49^ of each fitting component are displayed in the table.

	t1 (ps)	t2 (ps)
Hybrid_TE	174 ± 5 (88%)	600 ± 80 (12%)
Hybrid_TM	156 ± 5 (85%)	593 ± 60 (15%)
WS_2_	383 ± 30 (78%)	1040 ± 230 (22%)

In brief, the linearity of the PL at 750 nm results from the polarization-dependent plasmonic coupling leading to different degrees of enhancement in TE and TM polarizations. In contrast, the linearity of the PL at 640 nm is attributed to the geometrical birefringence of the nanowire array, which is represented as a high transmission of the TE polarization in the forward direction and a high reflection of the TM polarization in the backward direction. This is different from the wire grid polarizer whose period is much smaller than the tailored wavelength of light in which the geometrical birefringence is characterized as a high transmission of the TM polarization in the forward direction and high reflection of the TE polarization in the backward direction.

## Conclusion

In conclusion, we have demonstrated the strong anisotropic enhancement of the PL emission in WS_2_ integrated with the plasmonic nanowire array. The enhancement at different optical bandgap transitions stems from different mechanisms. For the indirect bandgap transition around 750 nm, multifold enhancement of the PL is attributed to the coupling of excitons with either the localized plasmon mode of metallic nanowires in the TM polarization direction or the plasmonic waveguide mode of the nanowire array on the dielectric substrate in the TE polarization direction. The localized mode I enables a higher degree of enhancement up to 7 because of a smaller mode volume and a stronger field localization around the surface of the metal wire. The waveguide mode II exhibits a spectral shift as a function of the period of the nanowire array allowing for the modulation of the excitonic emission in a wide spectral range. In contrast, for the direct bandgap transition, only a moderate enhancement of the PL (1–2.5x) in the TE polarization direction was observed which is probably due to the coherent superposition of the TE polarized light in the periodic array, just like the zero-order interference maximum in the diffraction grating. The linearity has a strong dependence on the wire width and periods of the array while it is less influenced by the polarization angle of the incident laser. Our study offers new opportunities for establishing ultrathin photonic devices with multi-functionalities which allows technical advancements in various important areas such as tunable displaying, ultrasensitive sensing, and encoded imaging.

## Supplementary information

Supplementary information

## Data Availability

*Scientific Reports* requires the inclusion of a data availability statement with all submitted manuscripts, as this journal requires authors to make available materials, data, and associated protocols to readers.
