# Supplementary material for: Strong anisotropic enhancement of photoluminescence in WS2 integrated with plasmonic nanowire array
Source: Sci Rep. 2021 May 12;11:10080. doi: 10.1038/s41598-021-89136-0 (PMC8115162; doi:10.1038/s41598-021-89136-0)
Supplement: Supplementary file 1 — Supplementary information [file 41598_2021_89136_MOESM1_ESM.docx]

**Supplementary Information**

**Strong anisotropic enhancement of photoluminescence in WS_2_ integrated with plasmonic nanowire array**

Chunrui Han^1,2^*, Yu Wang^1^, Weihu Zhou^1^, Minpeng Liang^2^, Jianting Ye^2^*

^1^Institute of Microelectronics, Chinese Academy of Sciences, Beijing 100029, China.

^2^Device Physics of Complex Materials, Zernike Institute for Advanced Materials, University of Groningen, Nijenborgh 4, 9747 AG, Groningen, The Netherlands.

* Corresponding authors. Email: [hanchunrui@ime.ac.cn](mailto:hanchunrui@ime.ac.cn); j. [ye@rug.nl](mailto:ye@rug.nl)

**1. Multicomponent fitting of Raman spectra**


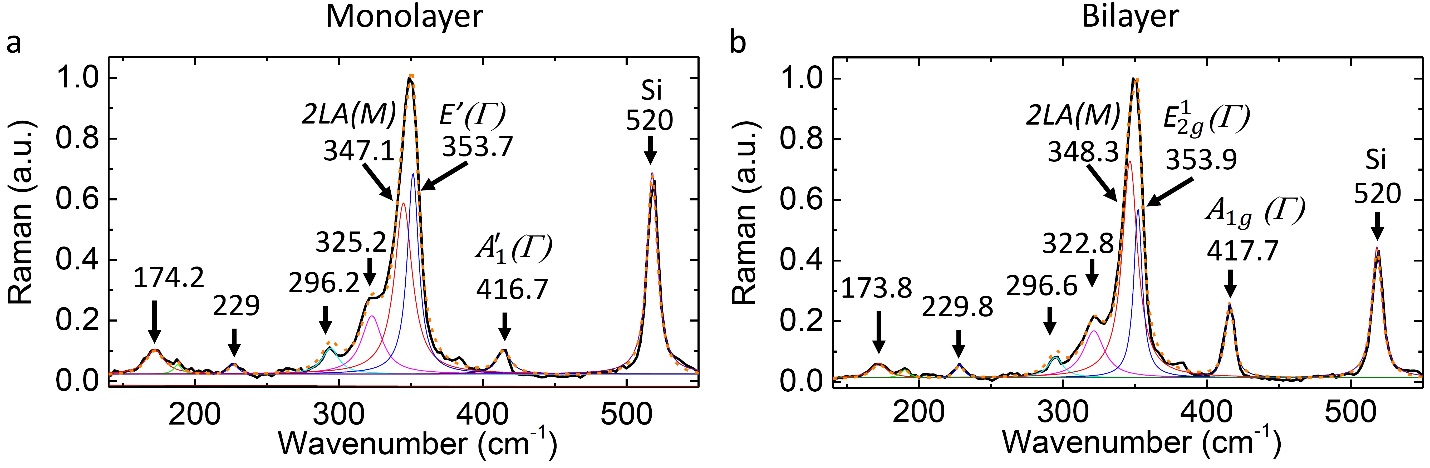


Figure S1 Raman spectra of mono- (a) and bi-layered (b) WS_2_ (Figure 1a in the main text) with 532 nm laser excitation including Lorentzian peak fitting. Black solid and orange dashed curves indicate the experimental and the fitted curves.

Multicomponent fittings of Raman spectra in Figure 1a in the main text are shown in Figure S1. The Raman peak around 350 cm^-1^ is typically labeled as *E*$'$(*Γ*) for an odd number of layers and $\text{E}_{2g}^{1}$(*Γ*) for an even number of layers^1^, which in fact includes two vibrational modes. For the monolayer, 2*LA*(*M*) and *E*$'$(*Γ*) modes are located at 347.1 and 353.7 cm^-1^, respectively. The intensity of 2*LA*(*M*) is ~ 5.7 times higher than that of $\text{A}_{1}^{'}$(*Γ*) mode. For the bilayer, 2*LA*(*M*) and $\text{E}_{2g}^{1}$(*Γ*) modes are located at 348.3 and 353.9 cm^-1^, respectively. The 2*LA*(*M*) mode is ~2.7 times higher than $\text{A}_{1g}$(*Γ*).

**2. Near field distributions of electric fields around the direct bandgap transition**

The near field distributions of electric fields at 636 nm are calculated by using FDTD numerical simulations as shown in Figure S2. For the TE polarization, we find that the electric fields penetrate the metallic wires and go to the far-field (Figure S2a-c), which is consistent with the high transmittance in the forward direction as shown in Figure 5a in the main text. It allows the coherent scattering of the TE polarized PL in the forward direction. The intensities of electric fields are quite similar for various wire widths, suggesting that the transmittance of the wire array (600 nm in period) has little dependence on the wire width. For the TM polarization, electric fields mainly locate below the nanowires whose intensities increase step by step with the wire width as shown in Figure S2d-f, suggesting that the reflectance in the backward direction increases with the wire width. This allows for the enhancement of the backward scattering of the PL in the TM polarization direction and hence the suppression of the emission in the forward direction. As a result, we can observe the enhancement/suppression of the direct bandgap transition in the TE/TM polarization direction as shown in Figure 4 in the main text.

**
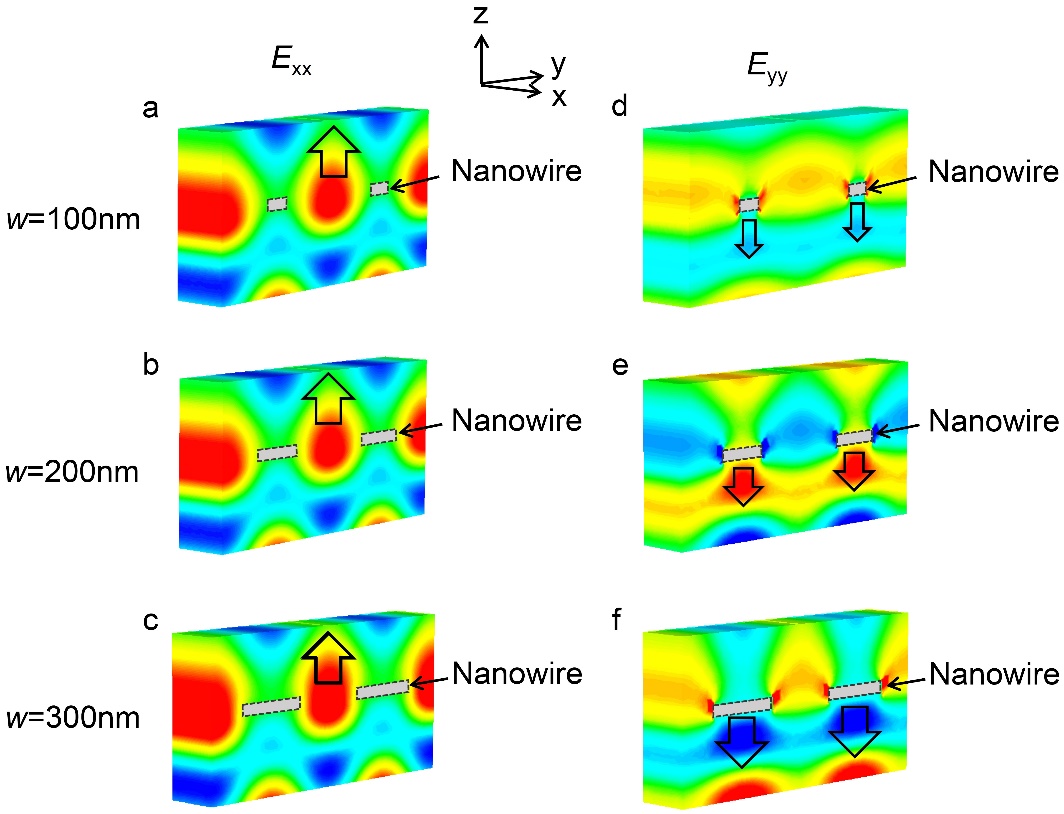
**

Figure S2 Electric field distributions of nanowire arrays on the Al_2_O_3_/SiO_2_ substrate with *p*=600 nm, *w*=100 (a, d), 200 (b, e), 300 (c, f) nm respectively for the TE (left column) and TM (right column) polarized light.

**3. Linearity as a function of the period of the nanowire array**

**3.1 Experiment**

The period of the nanowire array is a key parameter to tune the emission of the direct bandgap transition. It has been reported that the maximal enhancement of the radiative emission in vacuum locates at roughly *λ*~*p*/*m*, where *m* is an integer and *p* is the period of the array^2^. The enhancement arises from the coherent superposition of light waves passing through the nanowire array whose period is comparable with the wavelength of light. This is consistent with our observations in experiments, where the 600 nm wire array exhibits the maximal linear dichroism even for different filling factors, i.e. the ratio between the width and period *w*/*p* as shown by the red curves in Figure S3c and f. For *w*/*p*=0.25, the 600 nm period wire array enables the highest enhancement of the PL emission in the TE polarization direction leading to the maximum linear dichroism (red curve in Figure S3a, c). In contrast, the 400 nm period wire array has the lowest linear dichroism because the emission in the TM polarization direction is high (black curve in Figure S3b). For *w*/*p*=0.45, the PL intensity of the 800 nm period wire array is much lower than those of the 400 and 600 nm wire arrays (Figure S3d), resulting in the worst linearity among the three periods (blue curve in Figure S3f).


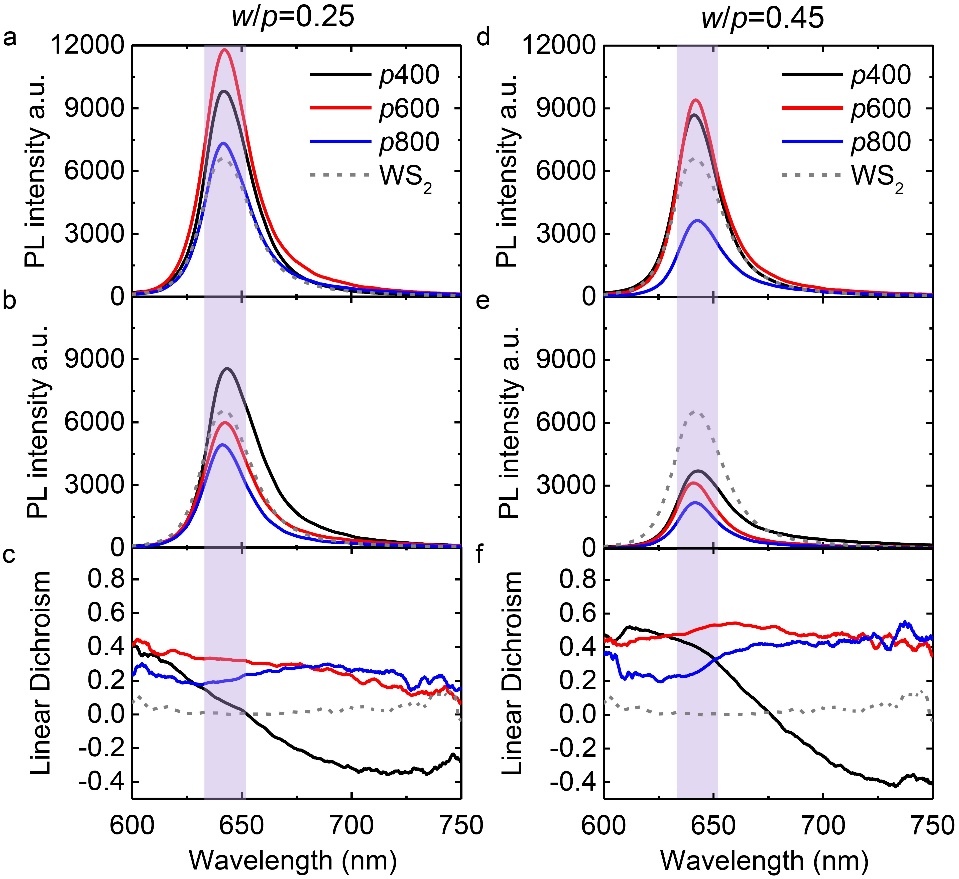


Figure S3 Anisotropic enhancement of the direct bandgap emission as a function of periods of the nanowire array. (a)-(b) PL spectra of the bare WS_2_ (dashed grey curve) and WS_2_-wire array hybrid structure with 400 (black curve), 600 (red curve), and 800 nm (blue curve) in the period, respectively, and filling factor *w*/*p*=0.25, for TE (a) and TM (b) polarizations. (c) The linearity of the WS_2_-nanowire hybrid nanostructure with *w*/*p*=0.25. (d)-(f) The corresponding results for nanowire arrays with filling factor *w*/*p*=0.45.

**3.2 Simulations for nanowire arrays on Al_2_O_3_ and Al_2_O_3_/SiO_2_**

To understand the emission behaviors of the WS_2_-nanowire array hybrid nanostructure with different periods, optical spectra of the nanowire array are calculated as shown in Figure S4. The forward transmittance in the TE polarization direction is the highest for the nanowire array of *p*=600 nm (red curves) on Al_2_O_3_ (Figure S4a, e) and Al_2_O_3_/SiO_2_ (Figure S4b, f) substrates, even for different filling factors, e.g. *w*/*p*=0.25 (Figure S4a, b) and 0.45 (Figure S4e, f). This enables the maximum forward scattering of the PL emission in the TE polarization direction among three periods (Figure S3a, d). The forward transmittance is the lowest for the nanowire array of *p*=800 nm (blues curves in Figure S4a, b, e, f) around the direct bandgap transition leading to the lowest PL intensity among the three periods as shown by the blue curves in Figure S3a, d. In particular, the PL emission is much lower for *w*/*p*=0.45 compared with that of *w*/*p*=0.25, leading to the minimum linear dichroism of the PL for the 800 nm period nanowire array (the blue curve in Figure S3f). The reflectance in the TM polarization direction increases dramatically when the filling factor *w*/*p* increases from 0.25 (Figure S4c, d) to 0.45 ((Figure S4g, h), hence the emission of the TM polarization in the forward direction decreases significantly as shown in Figure S3e, resulting in higher linear dichroism with larger filling factors (Figure S3f).


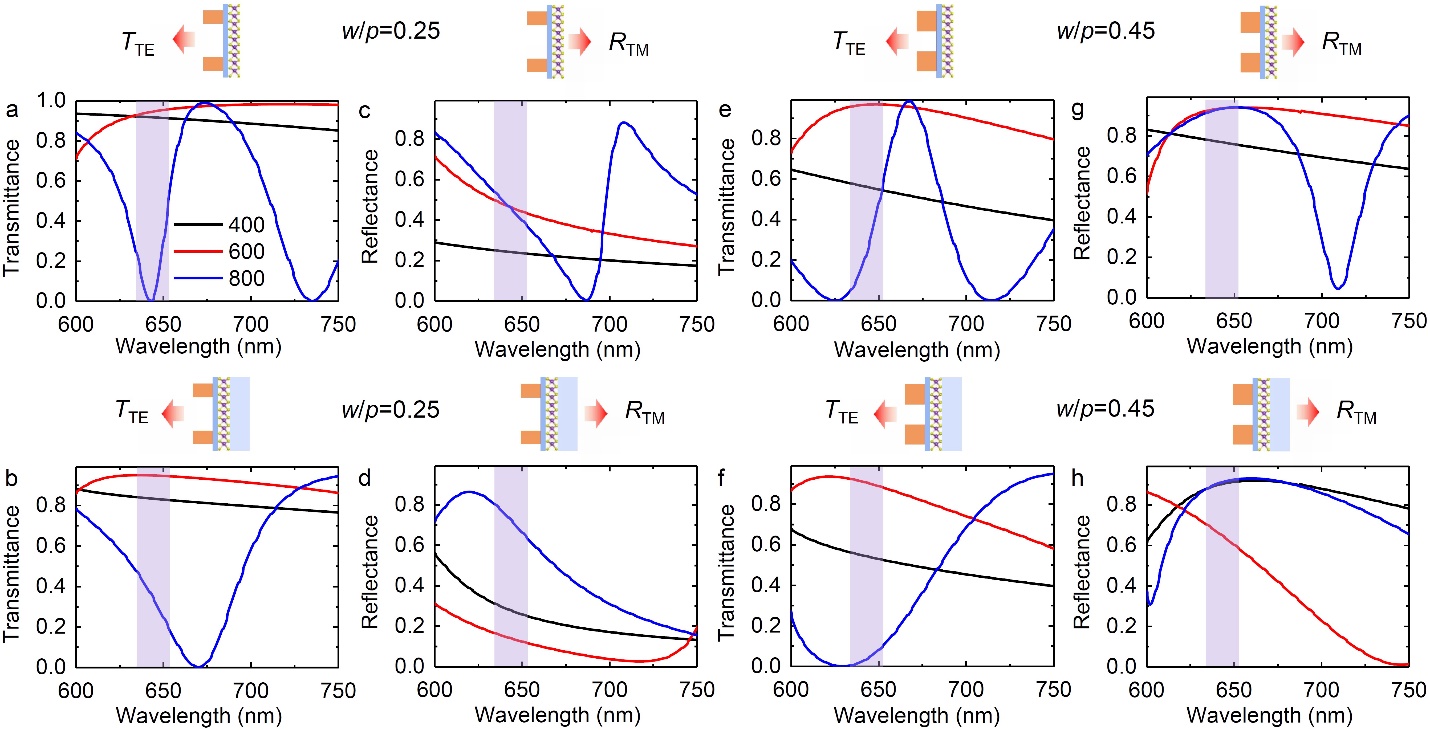


Figure S4 Polarization resolved spectra as a function of periods of the nanowire array by simulations. (a)-(b) Transmittance of the TE polarized light under the forward incidence for nanowire arrays on Al_2_O_3_ (a) and Al_2_O_3_/SiO_2_ (b) substrates with *p*=400 (black curve), 600 (red curve), 800 (blue curve) nm and *w*/*p=*0.25. (c)-(d) Reflectance of the TM polarized light under the forward incidence for nanowire arrays on Al_2_O_3_ (c) and Al_2_O_3_/SiO_2_ (d) substrates with *p*=400 (black curve), 600 (red curve), 800 (blue curve) nm and *w*/*p=*0.25. (e)-(h) Corresponding results for nanowire arrays with *w*/*p=*0.45.

**3.3 Simulations for nanowire arrays on Al_2_O_3_/SiO_2_/Si substrates**

For nanowire arrays on Al_2_O_3_/SiO_2_/Si substrates, we find that mode II (the same as mode II in Figure 3f in the main text) is red-shifted when the period of the nanowire array increases from 400, 500, 600, 700 to 800 nm. It suggests that the plasmonic modulation of the excitonic emission can be extended to a wide spectral range. Hence, various TMDCs with different optical bandgap transitions can be controlled by the plasmonic nanowire array.


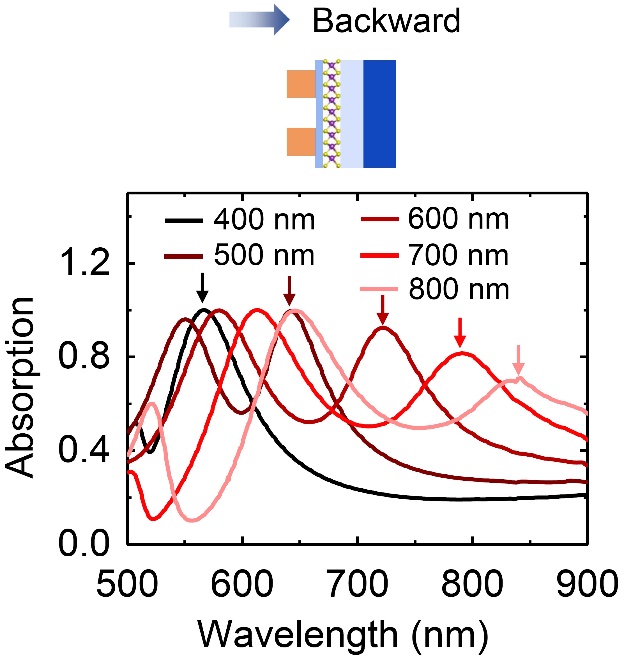


Figure S5 Absorption spectra of the TE polarized light under the backward incidence for nanowire arrays on Al_2_O_3_/SiO_2_/Si substrates with period *p*=400, 500, 600, 700, 800 nm and *w*/*p=*0.45 respectively.

**4. Characterizations of the sample for the time-resolved PL measurement**


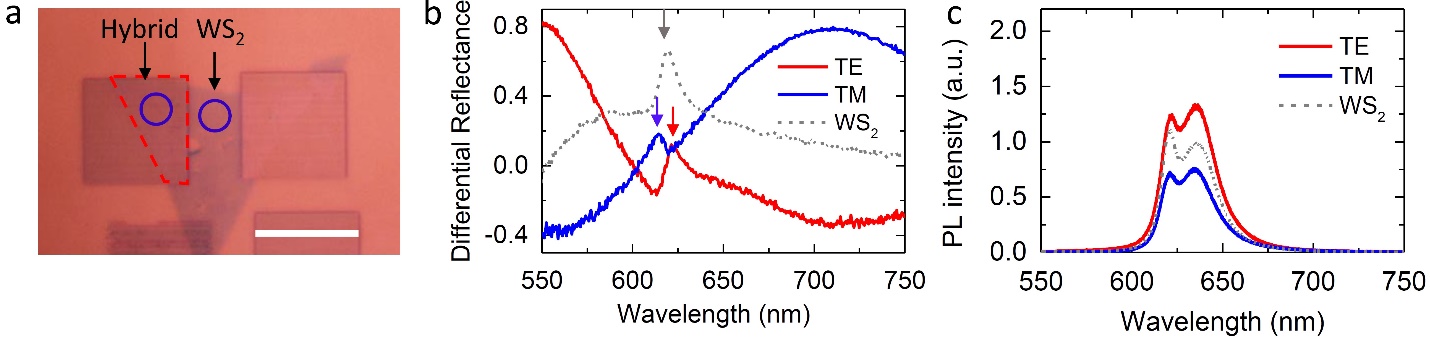
 Figure S6 (a) Optical image of the WS_2_-Ag wire hybrid nanostructure and the WS_2_ monolayer for the time-resolved PL measurement (Figure 8 in the main text). The positions for the measurement are indicated by the blue circles. Scale bar is 40 μm. (b)-(c) Differential reflectance and corresponding PL spectra for the bare WS_2_ (grey dashed curves), the hybrid nanostructure in TE (red solid curves), and TM (blue solid curves) polarization directions. Differential reflectance is calculated by (*R*_substrate_-*R*_target_)/*R*_substrate_.

An optical image of the measured sample is shown in Figure S6a. Blue circles indicate positions of the measurement for the time-resolved PL (Figure 8 in the main text), differential reflectance (Figure S6b) and PL (Figure S6c) spectra. Differential reflectance peaks represent the absorption of WS_2_ (grey arrow), WS_2_-Ag wire hybrid nanostructure in TE (red arrow) and TM (blue arrow) polarization directions. Positions of the peaks are different, probably due to the different changes of the dielectric environment in different polarization directions induced by the anisotropy of the nanowire array.

**5. Methods for materials growth and spectral measurement**

**Note 1: Materials growth.** Tungsten disulfide monolayers were synthesized via chemical vapor deposition. Tungsten triple oxide (99.995%) and sulfur (99.998%) powders were used as the source materials, silicon wafers with 285 nm SiO_2_ on top as substrates, and argon as carrying gas. Firstly, the quartz tube was flushed by a high flow rate of high-purity argon for 20 mins to remove the air completely. Then the argon flow rate was set as 100 standard-state cubic centimeter per minute (SCCM), and the furnace was rapidly heated to 850°C for tungsten triple oxide at 30 K/min and a separate heater heats the sulfur source to 190^o^C. This growth condition lasts for 10 minutes, followed by natural cooling down to room temperature.

**Note 2: Polarization resolved PL imaging measurement.** The sample was put on the sample stage and imaged by a 100x objective lens. WS_2_ flakes were excited by a 450 nm unpolarized light-emitting diode (LED). The PL was imaged by a CCD camera. The polarization of the PL emission was resolved by placing a polarizer before the CCD camera. The polarizer can be rotated to be either parallel or perpendicular to the long axis of the nanowire, or taken out of the optical path. As a result, the image of the TE-, TM- and non-polarized PL can be obtained by tuning the polarizer.

**Note 3: Polarization resolved PL/white light spectral measurement.** The excitation laser was 532 nm polarized in the TE direction; whose power was measured to be 104 μW under the 50× objective lens. The PL emission was collected by the spectrometer (Andor SR-500) and a CCD camera (iDus 420). The polarization of the PL emission is analyzed by placing a linear polarizer before the 532 nm edge filter and rotating it from zero to 360^o^ with 0^o^ (TE)/90^o^ (TM) defined as the angle parallel/perpendicular to the long axis of the nanowires. For white light spectral measurement, the polarization of the incident white light was controlled by placing a polarizer between the white light source and the sample, and then the reflected light was collected by the same spectrometer.

**Note 4: Time and polarization-resolved photoluminescence measurement. The** time- and polarization-resolved photoluminescence measurement was performed by using an Ultrafast micro-photoluminescence setup. The sample was excited by a picosecond pulsed diode laser with the excitation wavelength of 405 nm and a repetition rate of 80 MHz. The intensity and power density of the excitation laser are 240 nW and 76 mW/cm^2^. The polarization of the laser beam is parallel to the long axis of the nanowire. A 20x (NA=0.45) objective was used for the PL excitation and collection. The PL emission was resolved by a polarizer before entering the monochromator. TE and TM represent the PL polarization parallel and perpendicular to the long axis of the nanowire, respectively. The dynamics of the PL were measured using a single photon counting module (PicoQuant). The time resolution of the setup is ~40 ps. All measurements were conducted at room temperature.

**Note 5: The PL spectrum assignment.** In the main text, we assigned the low energy emission peak around 750 nm to the bilayer WS_2_, because the peak energy ~1.65eV coincides well with the indirect bandgap of bilayer WS_2_^3-5^. In the PL spectra, we also observed abnormally strong PL feature centered at ~ 640 nm, which seems to be contributed by monolayer emission. According to the literature reporting the CVD growth^3,6^, the monolayer growth starts from the nanometer-sized few-layer clusters. Hence, the present sample is likely to be composed of nano-islands of bilayer which are partially grown on a large monolayer flake. Such a mixed flake can explain the strong PL emission at 640 nm.

Note that there is another possible explanation for the simultaneous observation of 750 nm peak and strong PL at 640 nm, i.e. the latter is from monolayer and the former from defect modes. However, many kinds of defect modes (>1.85 eV) have been reported^1,4,7^ to be very close to the direct bandgap transition (1.95 eV), which are much larger than our case (1.65 eV). As a result, based on the peak energy, the bilayer scenario is more plausible.

**References:**

1. McCreary, A., Berkdemir, A., Wang, J., Nguyen, M. A., Elias, A. L. & Perea-López, N. Distinct photoluminescence and Raman spectroscopy signatures for identifying highly crystalline WS_2_ monolayers. *J. Mater. Res.* **31**, 931 (2016).
2. Pellegrini, G., Mattei, G. & Mazzoldi, P. Light extraction with dielectric nanoantenna arrays. *ACS Photonics* **3**, 2715–2721 (2009).
3. Gutiérrez, H., Perea-López, N., Elías, A., Berkdemir, A., Wang, B., Lv, R. *et al.* Extraordinary room-temperature photoluminescence in triangular WS_2_ monolayers. *Nano Lett.* **13**, 3447–3454 (2013).
4. Cong, C., Shang, J., Wang, Y. & Yu, T. Optical properties of 2D semiconductor WS_2_. *Adv. Optical Mater.* **6**, 1700767 (2018).
5. Yan, W., Meng, L., Meng, Z., Weng, Y., Kang, L. & Li, X. Probing angle-dependent interlayer coupling in twisted bilayer WS_2_. *J. Phys. Chem. C* **123**, 30684–30688 (2019).
6. Zhu, D., Shu, H., Jiang, F., Lv, D., Asokan, V., Omar, O. *et al.* Capture the growth kinetics of CVD growth of two dimensional MoS_2_, *npj 2D Mater. Appl.* **1**, 1–8 (2017).
7. Wang, X., Dan, J., Hu, Z., Leong, J., Zhang, Q. *et al.* Defect heterogeneity in monolayer WS_2_ unveiled by work function variance. *Chem. Mater.* **31**, 7970−7978 (2019).
